# In vivo safety assessment of a bio-inspired bone adhesive

**DOI:** 10.1007/s10856-020-6362-3

**Published:** 2020-02-08

**Authors:** Gry Hulsart-Billström, Christina Stelzl, Philip Procter, Michael Pujari-Palmer, Gerard Insley, Håkan Engqvist, Sune Larsson

**Affiliations:** 1 Division of Orthopaedics, Department of Surgical Sciences, Uppsala University, Uppsala, 751 85 Sweden; 2 Division of Applied Material Science, Department of Engineering Sciences, Uppsala University, Uppsala, 751 21 Sweden; 3 GPBio Ltd, Unit 4D, Western Business Park, Shannnon, Co. Clare Ireland

## Abstract

A new class of materials, bone adhesives, could revolutionise the treatment of highly fragmented fractures. We present the first biological safety investigation of a bio-inspired bone adhesive. The formulation was based upon a modified calcium phosphate cement that included the amino acid phosphoserine. This material has recently been described as substantially stronger than other bioresorbable calcium phosphate cements. Four adhesive groups with the active substance (phosphoserine) and two control groups without phosphoserine were selected for in vitro and in vivo biocompatibility testing. The test groups were subject for cell viability assay and subcutaneous implantation in rats that was followed by gene expression analysis and histology assessment after 6 and 12 weeks. All adhesive groups supported the same rate of cell proliferation compared to the α-TCP control and had viability between 45–64% when compared to cell control. There was no evidence of an increased immune response or ectopic bone formation in vivo. To conclude, this bio-inspired bone adhesive has been proven to be safe, in the present study, without any harmful effects on the surrounding soft tissue.

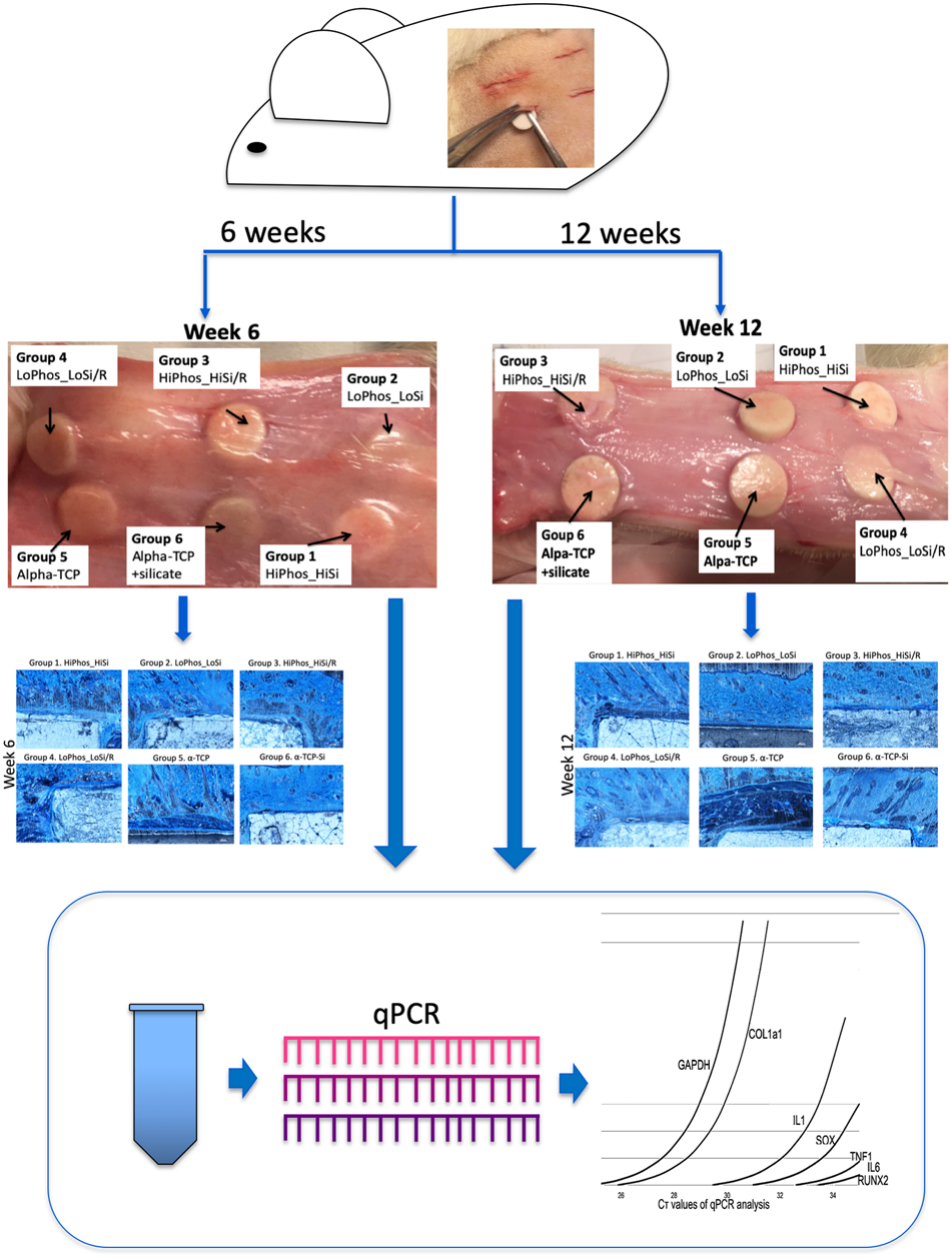

## Introduction

Due to bone tissue’s excellent healing properties fixation using a cast or metal hardware is, in general, a suitable fracture treatment post-traumatic injury. In certain fractures, where there are many small bone fragments or the fracture is adjacent to joints, metal implants—such as screws, pins, plates and intramedullary nails—may be a suboptimal treatment. Bone adhesives could revolutionise treatment in these cases by (1) reducing the amount of metal hardware necessary to stabilise small fragments, (2) reduce surgery time and (3) obviate the need for secondary surgery to remove metal hardware. The net result would be a reduction in cost and improvement in patient safety. Despite a clear clinical need, and despite many attempts to develop such biomaterials, effective bone adhesives are still not available on the market. The main hurdles for this class of material are the numerous and complex properties that need to be fulfilled in the environment of healing bone. Heiss et al. [1] and Farrar [2] highlight the difficulties and desired properties needed for bone adhesives [1, 2]. In short, a successful bone adhesive needs to be biocompatible, display adequate bond strength, form adhesive bonds in wet and fatty environments and have simple handling procedures to facilitate surgery. In recent years, research on biological inspired adhesives for wet field applications has focused on adhesives inspired by marine animals, such as mussels and sandcastle worms [3–6]. The adhesive molecules derived from such marine animals, typically a complex protein or coacervate, have yet to be shown to be either safe or effective in the bonding and subsequent healing of human tissues. In this paper, we present the first biological safety investigation of a new class of bio-inspired bone adhesive [7–11].

The formulation investigated, herein, is a modified calcium phosphate cement (CPC) that included the amino acid phosphoserine. Phosphoserine modified cements (PM-CPC) have recently been described as substantially stronger than other bioresorbable calcium phosphate cements [7, 9]. PM-CPCs produce strong adhesion to tissues and biomaterials, producing 40-fold stronger adhesion strength (1–4 MPa), compared to the reported strength of cyanoacrylate, under wet-field conditions. In an ex vivo rat model of bone reconstruction, using a metaphyseal bone defect, the bond (failure) strength was 30-fold higher than a commercial surgical adhesive (fibrin glue, Tisseel) [9]. The reported failure mode of PM-CPC occurred in the tissue rather than the adhesive. While Tisseel failure was soft (low modulus), resembling deformation by creep, PM-CPC failed abruptly, with several of the samples fracturing in the bone plug rather than in the adhesive. The mechanism underlying the strong adhesiveness of PM-CPCs has recently been explored by Pujari-Palmer et al. and Spicer et al. [7, 10]. They postulated that the nanoscale interactions between phosphoserine and the amorphous calcium phosphate, combined with the hierarchical organisation of the organic phase of the adhesive, increased the interaction surface area (physical bonding) and electrostatic interactions (Van der Waals bonding), thereby creating strong adhesion to surfaces. Spicer et al. [10] has also shown that bidentate calcium-binding motifs, together with stereospecific orientation, and substrate hydrophobicity, all play a crucial role in the final adhesive strength. Collectively, these prior reports have shown that CPCs can be transformed into adhesives by a broad spectrum of heterobifunctional organophosphates. Interestingly, the same adhesive effect has also been demonstrated in many different types of tissues, including soft tissues, skin, cartilage, heart, and muscle [11]. An adhesive PM-CPC formulation, similar to the composition investigated in the present study, has also exhibited excellent osteointegration, and histocompatibility, for up to 1 year, in a condyle rabbit model [8].

In the present study an alternative calcium salt, calcium silicate, was included at both low (vol/wt 2%) and high amounts (28%). Calcium silicate has been reported, in a recent systematic review, to be a beneficial compound for bone healing [12].

CPCs have been used as a bone substitute, most often as bone void fillers, as well as in a variety of tissue engineering applications. Generally, they are considered to be biocompatible [13]. Nonetheless, changes in composition can significantly impact the material properties, including biocompatibility, cell adhesion, osteoconductivity and osteoinductivity. The influence of phosphoserine on these properties is not yet fully understood, with conflicting results reported in the literature, as the following findings show. Initially, cell proliferation was reported to be stimulated in cells that were seeded on modified calcium phosphate cements (containing collagen and phosphoserine) [14, 15]. However, cell media containing phosphoserine had no impact on cell proliferation [16, 17]. This is a good example of the complex interaction between cells or tissues and phosphoserine containing materials. Importantly, phosphoserine has been reported to have a significant impact on cell differentiation towards the osteoblastic lineage [15, 16, 18]. Phosphoserine appears to produce higher ALP activity [16], higher collagen synthesis rate [14] and increased calcium deposition [17]. It is important to note that these studies were mainly conducted on CPCs modified with collagen, so the results are confounded by effects of the collagen content on adhesion and proliferation rate.

CPCs modified with phosphoserine can contribute to improved bone healing capacities [13]. Phosphoserine soaked hyaluronic acid produced significantly more bone formation in a rabbit defect model, than an analogue lacking phosphoserine [16]. Similarly, CPCs with phosphoserine led to higher bone remodelling and bone formation in rats [19]. It has also been reported that phosphoserine accelerates the resorption of calcium phosphate cements in mini pigs [20].

The objective of the present study was to provide proof of safety and biocompatibility on PM-CPCs, in vitro and in vivo. Four different PM-CPCs were tested with different concentrations and combinations of the active substance and silicate (high versus low). Biosafety was examined via cell viability, and histological assessment. For in vivo assessment 18 rats received subcutaneous implants for 6 and 12 weeks respectively. The subcutaneous implants were subject to histology, and gene expression analysis via RNA extraction.

## Materials and methods

### Material production

For the subcutaneous implants, six different formulations of PM-CPCs were selected. The material compositions are summarised in Table 1. The base materials were purchased from Aalborg (calcium silicate, Portland cement), Flamma S.p.A Italy (Phosphoserine, >95%), or produced as described previously (α–TCP, >95% [7]). In short, four adhesive groups with the active substance (phosphoserine) and two control groups without the active substance were fabricated as follows: after mixing at the following weight ratios (HiPhos_HiSi, LoPhos_LoSi, etc., as seen in Table 1), discs 8 × 3 mm were cured for 24 h, polished to 2 mm thickness to remove surface defects, and sterilised through gamma radiation (PBC Biomed Ltd, Ireland). For cell viability testing the discs were prepared similarly using 3 × 8 mm discs but without additional polishing step (Table 1).

**Table 1:** Material composition for the 6 calcium phosphate cement groups and respective liquid phase

Group number	Powder phase	Liquid phase
Test groups		
1 HiPhos_HiSi	45% phosphoserine, 28% silicate	Water
2 LoPhos_LoSi	25% phosphoserine, 2% silicate	Water
3 HiPhos_HiSi/R	45% phosphoserine, 28% silicate	30% w/v NaCa
4 LoPhos_LoSi/R	25% phosphoserine, 2% silicate	30% w/v NaCa
Controls		
5 α-TCP	αTCP	2.5% Na_2_HPO_4_
6 α-TCP-Si	αTCP, 28% silicate	2.5% Na_2_HPO_4_

**Table 2:** Primer list for gene expression assay, TaqMan assay (Applied Biosystems, Foster City, USA)

Gene name	Symbol	TaqMan® assay
Collagen type I alpha 1 chain	Col1a1	Rn01463848_m1
Tumour necrosis factor	Tnf	Rn99999017_m1
Runt-related transcription factor 2	Runx2	Rn01512298_m1
Interleukin 1 beta	Il1b	Rn00580432_m1
Interleukin 6	Il6	Rn01410330_m1
SRY box 9	Sox9	Rn01751070_m1
Glyceraldehyde-3-phosphate dehydrogenase	Gapdh	Rn01775763_g1

### Surgical procedure

**Fig. 1:**
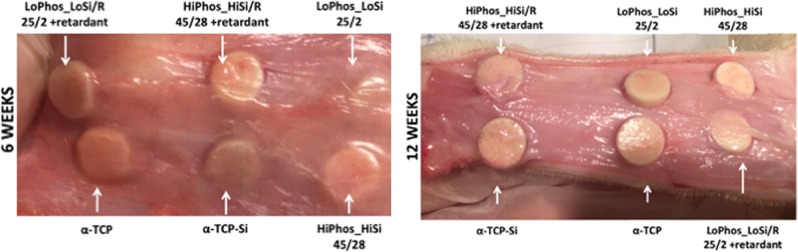
Macroscopic images of the explants after 6 and 12 weeks. There were no traces of inflammation or fibrotic tissue surrounding the samples. Small blood vessels were distributed across the connective tissue without any disruption or bleeding. The connective tissue was not integrated into the material and moved smoothly over the materials disks

The animal study was approved by the Uppsala Committee of Animal Research Ethics (5.8.18-11832/2017), according to the Federation of European Laboratory Animal Science Association’s guidelines (European Directive 2010/63/EU). The surgeries were performed under aseptic conditions. A total of 18 healthy male Sprague Dawley rats were used in this experiment and 9 rats were allocated for each time point. The rats (Taconic Farms Inc., Silkeborg, Denmark) were weighed (400–450 g) and each then anaesthetised in an induction chamber with 5% isoflurane (Baxter, Sweden), 0.3 l/min oxygen. The rats were transferred to an anaesthesia mask with reduced isoflurane (1–2.5%), 1.0 l/min oxygen and placed on a heated pad during surgery. The back of each animal was shaved and disinfected with 5 mg/ml chlorhexidine (Fresenius Kabi, Uppsala, Sweden). In order to allow paired observations, each animal received six subcutaneous implantations, one from each group, with a volume of 0.2 ml. For each implantation, a 10–12 mm-long skin incision was made at a consistent position on the back of each animal, after which the respective disc was placed inside the subcutaneous pouch [21, 22]. The skin was sutured with resorbable 4–0 sutures (Vicryl, 4-0, C-3 needle, Ethicon, Agnthos, Sweden). Post-operative doses (3 days) of 0.05 mg/kg buprenorphine were administered subcutaneous for analgesia. The animals were allowed to move freely after surgery in Makrolon 4 cages (two rats/cage) with elevated lid and enrichment such as tree houses and bedding material with ad libitum of food pellets and water. After 6 and 12 weeks, the rats were anaesthetised in 2.5% isoflurane and euthanised in a CO_2_ chamber (Makrolon III cage with dimensions 382 × 220 × 150 mm). The cycle of the CO_2_ chamber was 50% oxygen and 50% CO_2_ for 6 min, followed by 100% CO_2_ for 6 min and then a second cycle of 2 × 6 min of CO_2_, after which the rats were confirmed dead by decapitation and the implants were then explanted (Fig. 1).

### Cell culture

MC3T3, osteoblast precursor cells, were cultured in DMEM/f12 media (supplemented with 10% FBS, 1% Penicillin-streptomycin; Gibco, Carlsbad, USA) in a standard incubator (37 °C, 5% CO_2_) and passaged before confluence.

### Presto Blue cell viability assay

The Presto Blue™ cell viability assay (Invitrogen, Carlsbad, USA) was performed 24 h after seeding, and each material group was used in triplicates. A cell control was used with the respective cell density on tissue culture plastic and a material control without seeded cells was included to exclude background measurements.

The sterile material discs were washed once with 1x PBS. Then seeding was performed with a cell density of 50,000 cells/cm^2^. After 4 h of incubation the material discs were moved to a new 48 well plate and fresh media was replenished on all samples (DMEM/f12; 10% FBS, 1% PenStrep;Gibco, Carlsbad, USA).

After the incubation period, the media was replaced with media containing 10% Presto Blue™ reagent and incubated until a colour change could be observed. Then 100 µl of cell media was used to measure fluorescence with excitation of 530–560 nm and emission of 590 nm.

### Analysis

Initial data analysis was performed in Microsoft Excel. Grubbs outlier test was performed to exclude significant outliers using Prism (Graph Pad Software Inc., USA). Subsequently, data was statistically analysed in SigmaPlot (Systat Software, San Jose, CA.) through Kruskal-Wallis One Way ANOVA on Ranks followed by Tukey’s Multiple Comparison test (chosen significance level *p* ≤ 0.01) to test all groups against each other. Data is presented as percentage of cell viability compared to cell control.

### Gene expression

Tissue samples were obtained from three rats per time point, at 6 weeks and 12 weeks post implantation. A small tissue sample was removed from subcutaneous tissue surrounding each implant. and stored in RNALater (Invitrogen) at −20 °C. Total RNA was extracted with TRIzol (Invitrogen, Carlsbad, USA) according to standard protocol. In short, tissue samples were homogenised in TRIzol reagent (Invitrogen). Chloroform was added which leads to a phase separation where RNA remains in the aqueous phase. This phase was used for subsequent RNA purification. Extracted RNA was quality controlled using NanoDrop 2000 spectrophotometer (Thermo Fisher Scientific, Carlsbad, USA) and diluted to a concentration of 10 ng/µl with RNase free milli-Q water.

Subsequently, cDNA synthesis was performed with High-Capacity RNA-to-cDNA™ Kit (Applied Biosystems, Foster City, USA) and further diluted 1:2 for subsequent qPCR run. For qPCR setup a reaction mix of TaqMan® Fast Universal Master Mix (2×), No AmpErase® UNG (Applied Biosystems, Foster City, USA) with TaqMan assay for the specific gene (Table 2) and cDNA was assembled to a total reaction volume of 20 µl. The assays were performed in technical triplicates. qPCR was run using the 7500 Fast Real-Time PCR System, with 40 cycles of standard thermal profile (95 °C 00:01 and 60 °C 0:20).

### Analysis of qPCR data

Analysis of qPCR data was performed using 2-ΔΔcT method in Microsoft Excel. Grubbs outlier test was performed in GraphPad prism (GraphPad Software, California) and outliers were removed from further analysis. For week 6, one outlier each was removed from the dataset of IL1 and SOX9 while two outliers were removed from the IL6 dataset. For week 12, one outlier was removed from the datasets of Il1, IL6 and SOX9, respectively.

### Histology

The explants that were examined by gene expression were later subjected to histological examination. The explants were fixed in 4% paraformaldehyde and then decalcified in modified formic acid in a Decalcifier System (Sakura Finetek Europe, Netherlands). They were later dehydrated in ethanol series, after which they were embedded in paraffin. Histological Section (7 µm) were prepared (Microtom Int, Walldorf, Germany), deparaffinised, hydrated, and stained with hematoxylin-eosin to assess the tissue reaction to the material. Another set of samples from both time points where dehydrated and infiltrated, after which they were embedded in resin (Technovit 7200) and grinded to 30 µm slides (Exakt Instruments, Hamburg, Germany). The slides where later stained in Richardson’s stain and examined by light microscopy (Leica). All chemicals where purchased from Histolabs Products AB (Gothenburg, Sweden).

## Results

### Cell viability

**Fig. 2:**
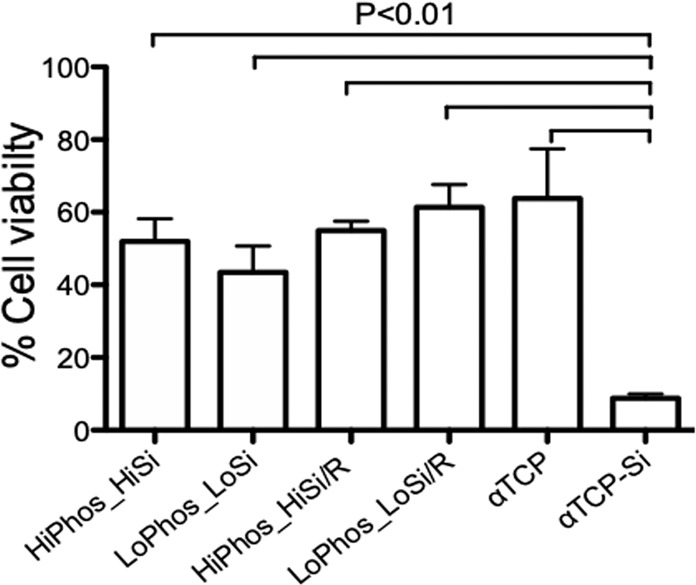
Presto Blue™ cell viability assay after 24 h incubation. MC3T3 cells seeded on 6 groups of gamma radiated materials. Cell viability after 24 h incubation expressed as percentage of cell control viability. The data was obtained from pooled data from 5 samples/groups tested in independent triplicates. Statistically significant differences in cell viability were found between α-TCP-Si and the other groups, no statistical differences were found between the adhesive groups and α-TCP control (One-way ANOVA, Tukey’s Multiple Comparison Test = *P* < 0.01)

Cell viability was assessed using Presto Blue cell viability assay. All adhesive test groups supported the same rate of cell proliferation compared to the α-TCP control (Fig. 2) but showed statistical significance to the silicate containing control group with lower than 10% cell viability. The viability for all other groups was between 45 and 64% compared to cell control.

### Macroscopic examination

There was no sign of redness or swelling the days after the implantation and all the wounds healed without any complications. The macroscopic examination showed no sign of inflammation or fibrotic tissue surrounding the samples. The connective tissue with small blood vessels demonstrated no signs of disruption or bleeding. The tissue moved smoothly across the materials disks, indicating no or low integration with the material.

### Gene expression

**Fig. 3:**
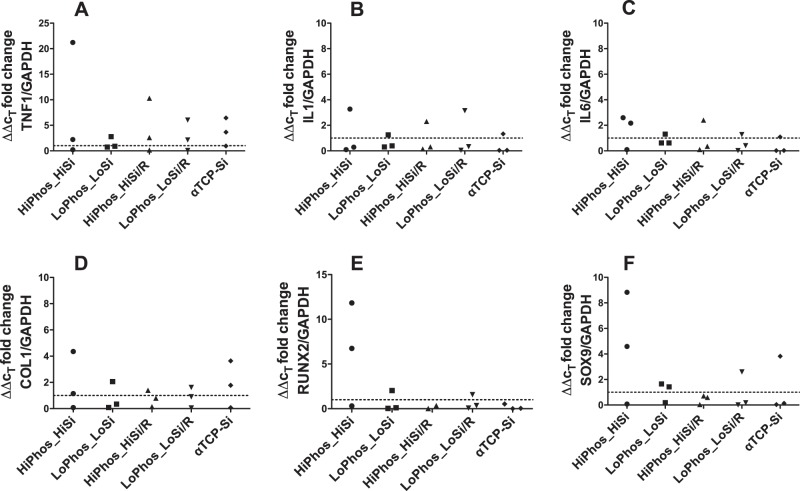
qPCR analysis of gene expression changes in response to in vivo subcutaneous implantation of bone adhesive. Tissue samples of three rats 6 weeks after implantation of the six material groups. qPCR performed in triplicates, normalised against GAPDH housekeeping gene and 2-ΔΔCt calculated in comparison to alphaTCP control. Dotted line represents α-TCP control as baseline for comparison

**Fig. 4:**
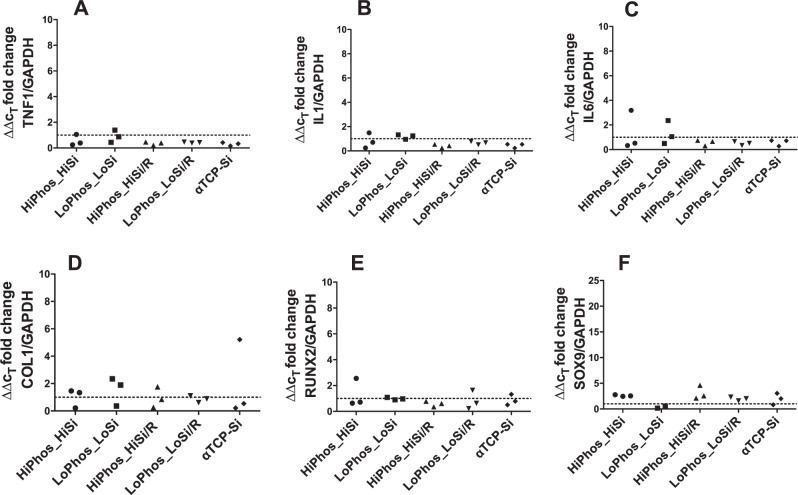
qPCR analysis of gene expression changes in response to in vivo subcutaneous implantation of bone adhesive. Tissue samples of three rats 12 weeks after implantation of the six material groups. qPCR performed in triplicates, normalised against GAPDH housekeeping gene and 2-ΔΔCt calculated in comparison to alphaTCP control. Dotted line represents α-TCP control as baseline for comparison

Gene expression analysis through qPCR was performed, at 6- and 12-weeks post implantation, to study whether the 4 different adhesive formulations and the two control groups elicit a different gene expression in vivo (summarised in Figs 3 and 4). Two groups of genes were investigated: firstly, bone marker genes Col1a1, SOX9 and RUNX2 and secondly, the immune marker genes, IL1, IL6 and TNFa. A high number of cycles, were used in order to detect any signal from the gene of interest except for the Col1a1. Both gene groups showed high variation in expression with no significant difference between the test materials and the α- TCP control. A result from low expression of the genes in the tissue surrounding the samples. The low signal for the genes of interest in all samples indicates that PM-CPCs do not elicit any unexpected immune or tissue responses, compared to the positive α-TCP control.

### Histology

**Fig. 5:**
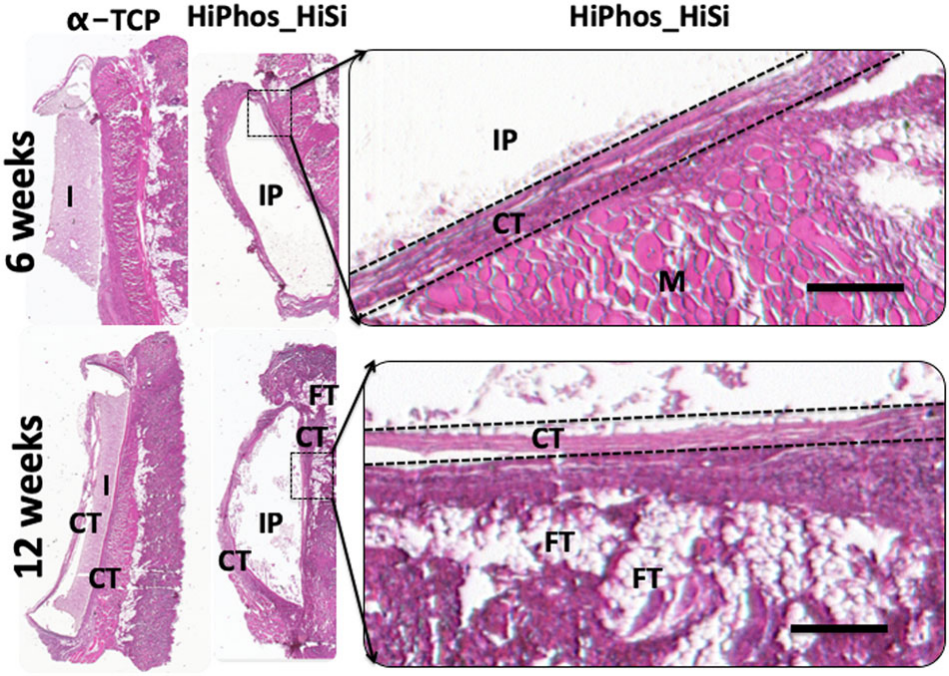
Hematoxylin Eosin staining of the positive control and HiPhos_HiSi. There was no sign of adverse effects in the interface between the materials and the normal layers of undisturbed connective tissue. There was a low amount of cells presents which is shown by the low amount of nuclei stained by the hematoxylin (dark blue stain). No sign of multinuclear cells and the dermal tissue was unaffected by the underlying implant (white pocket). The materials stayed inert and did not induce any tissue differentiation or fibrotic capsules. I implant, IP implant pocket, CT connective tissue, FT fat tissue, M muscle

**Fig. 6:**
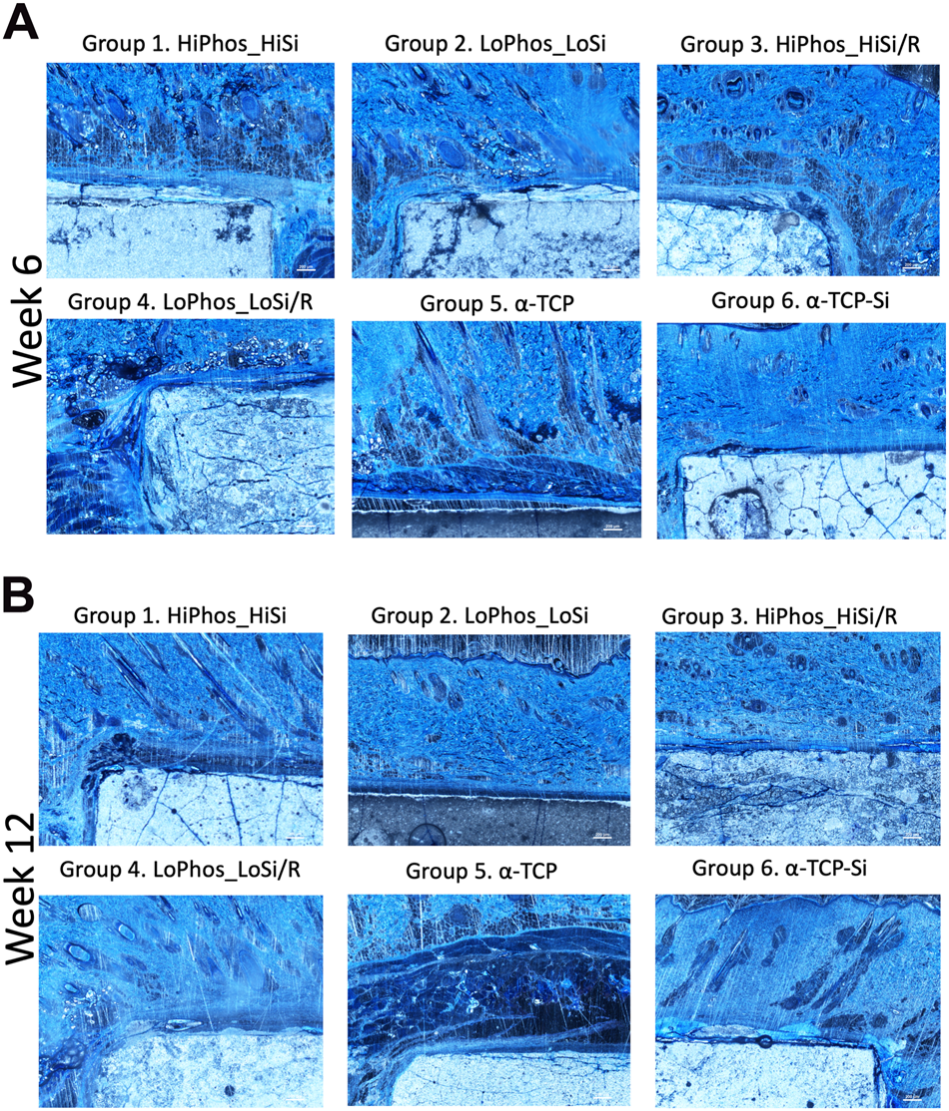
Richardson’s staining of all samples at (**a**) week 6 and 12 weeks (**b**). There was no sign of integration between the samples and the materials. Internal cracks and aggregations where present in all samples

The histology sections depicted no adverse reaction to the material, with mainly connective tissue (pink tissue) and a low density of cell nuclei (dark blue stain). All materials displayed no adverse reactions, comparable to the positive control of α-TCP. In addition, there were no signs of adverse effects at the interface between the materials and the normal layers of connective tissue. No multinuclear cells were observed and the dermal tissue was unaffected by the underlying implant (white pocket). The materials remained inert and did not induce any tissue response or fibrotic capsules (Figs 5 and 6).

## Discussion

The adhesive neither triggered an increased immune response nor ectopic bone formation: a desirable characteristic of a biomaterial tailored to glue bone fragments and guide bone conduction to promote full regeneration.

Implantation of foreign material can lead to unwanted responses in the body. More drastic reactions, such as outright implant rejection and severe inflammation, are easy to discern in vivo. In the present study the macroscopic findings in vivo did not involve an adverse immune reaction. However, more subtle reactions may be harder to detect. Thus, gene expression analyses were performed to elucidate underlying effects and safety of the bone adhesive, i.e. whether the different material formulations elicited a gene response in the surrounding tissue of the implant.

For this study two different groups of genes were chosen. Firstly, three genes (Il1b, Il6 and TNF), for which expression is linked to inflammatory responses [23–25], and secondly, three bone marker genes (Col1a1, Sox9 and Runx2), [26, 27], were analysed. The combination of the two groups of gene types allowed the screening of both inflammation, and regeneration, with respect to bone induction. There was no distinctive effect on the tissue from any of the material groups. The inert property of the material in soft tissue is a desirable quality, as a safe biomaterial for bone should not induce any activity at ectopic sites. There was no evident difference observed at the two time points, when the four adhesive groups and a control group with a high silicate content were compared to the α- TCP control. It should be noted that all genes of interest had a very low expression level compared to the house keeping gene and a high variation between the biological replicates was observable. This reduces the significance of the fold changes. Again, verifying the lack of any tissue response on the gene expression level. Collectively, the gene expression data effectively demonstrate that PM-CPCs do not produce aberrant immune or tissue responses in vivo and should, therefore, be considered safe for further, longer term implantation studies. The gene expression results were in correspondence with the macroscopic examination and the histological assessment, which both confirmed the inert nature of the adhesive when distributed in subcutaneous tissue. The surrounding connective tissue demonstrated native characteristics with the absence of any effect induced from the materials.

It was previously reported that phosphoserine causes an increase in bone marker gene expression [17]. However, considering the material discs were implanted subcutaneously a strong ectopic expression of bone marker genes would be unexpected. Two endpoints were chosen, the six-week time point allowed for the investigation of the material effect rather than the response to the surgical incision. The later time point of 12 weeks allowed for the possibility of long-term rejection of the material and formation of fibrotic tissue.

A related bone adhesive was tested in a rabbit condyle defect model, demonstrating a degradation of the material without any adverse effects after one year. Even though the material was tested without controls, and with four animals per time point, the data supports further testing and exhibited a corresponding result of “no adverse effects” [8]. Future work from our group will investigate the impact of phosphoserine-containing adhesives, PM-CPCs, on bone marker gene expression in a bone inductive setting. However, the absence of an immune marker gene induction is a strong argument supporting the biosafety of this novel biomaterial. Considering the lack of bone gene up-regulation, the obvious conclusion is that implanted phosphoserine modified calcium phosphate cements did not induce a material-specific response in the subcutaneous tissue.

Comparable rates of cell viability were observed when comparing the adhesive groups and the pure α-TCP control. The α-TCP-Si group showed decreased viability, likely due to very alkaline pH caused by calcium silicate that was neutralised by the strong acidity of phosphoserine in the adhesive material groups. It is also possible that inclusion of calcium silicate affected the setting and subsequent ion release rate of the control group, but not phosphoserine containing materials. Both pH and ion release are critical parameters that affect cell viability in vitro, but have much less impact in vivo. Interestingly, the in vitro findings did not mirror the in vivo results from the same material groups, supporting the assertion that pH and ion release effects could be the cause of lower than expected cell viability. In many studies the pH is neutralised before the material is introduced to cell culture or in vivo implantation. While this can be suitable in some set-ups, it can mask the real action of the material in situ. In this case the application of this material would be an injectable CPC that is hardening in situ. Therefore, the discs were not neutralised prior to any of the assays. For example, the indication of the alkaline calcium silicate being neutralised by the strong acidity of phosphoserine in the adhesive material groups, would not have been observed. Furthermore, different adherence properties such as different nano structures of the material surface could have introduced a higher variation and could mask less striking differences. These possibilities will be further studied in future experiments.

The collective findings of the present study suggest that PM-CPC adhesive formulations can be considered biocompatible, as they have comparable performance to the positive control material (αTCP) throughout all the assessments. There is no evidence, neither on macroscopic histological nor gene expression level, to suggest that the adhesive would be harmful in vivo. These findings encourage further investigation of the material, concurrent with on-going development of an in vitro adhesiveness test to assess the biological performance of the adhesive. The future application of the adhesive will be as an in situ curing bone adhesive. Any toxicity from the curing process as well as additional work of the potential osteoconductive behaviour is under investigation and will be reported in future studies.

## Conclusion

We have developed and evaluated a bio-inspired bone adhesive that has proven to be safe, in the present study, without any harmful effects on the surrounding soft tissue. The initial safety profile of this material, with the full absence of any harmful reaction, warrant further investigation of the material to fully explore the potential and function of the adhesive in vitro and in vivo, in novel orthotopic models.
